# The 4YouLab Model: A Dedicated-Program for Adolescents and Young Adults With Cancer in a Children’s Hospital

**DOI:** 10.3389/fonc.2021.705419

**Published:** 2021-07-01

**Authors:** Andrea De Salvo, Maria Antonietta De Ioris, Domitilla Secco, Francesca Bevilacqua, Roberto Premuselli, Matteo Amicucci, Italo Ciaralli, Francesca Santato, Angela Mastronuzzi, Giuseppe Maria Milano

**Affiliations:** ^1^ Department of Hematology/Oncology, Cell and Gene Therapy, Bambino Gesù Children’s Hospital, Istituto di Ricovero e Cura a Carattere Scientifico (IRCSS), Rome, Italy; ^2^ Unit of Clinical Psychology, Department of Neuroscience and Neurorehabilitation, Bambino Gesù Children’s Hospital, Istituto di Ricovero e Cura a Carattere Scientifico (IRCSS), Rome, Italy

**Keywords:** ca****ncer, AYA, adolescents and young adults, hospital care, oncology—discipline

## Introduction

Recently, there has been a growing attention to patients defined by the acronym AYA (adolescents and young adults). Therapeutic strategies and management protocols were developed to recognize the specific psychosocial needs of this age group (1, 2).

A cancer diagnosis places the patients at risk of adaptation disorders and anxiety-depressive syndromes due to emotionally and psychologically impacting effects (3). The effects can be more severe in adolescents (4). The disease and its treatment could compromise adolescent developmental paths: the construction and affirmation of the identity, the autonomy development, and the independence from the family unit (5, 6).

Currently, the patients who need to be treated go beyond the oncological cure, utilizing a biopsychosocial approach involving the patients’ personal and social norms. One of the strategies for a specific professional care in the multidisciplinary team is to ensure an adequate quality of life during hospitalization (6). From this point of view, it is essential to support discussion and aggregation with other adolescents in the hospital setting, reduce isolation, and encourage emotional sharing and expression. By organizing moments for aggregation, sharing is encouraged between patients, which is helpful to the patients’ well-being and quality of life during treatment.

The AYA cancer team utilizes a multidisciplinary care model focused on providing each patient with a holistic and tailored approach including social support together with the best cancer treatments. This approach is more attentive to quality of life and encourages the peer support group to “engage in conversation about having cancer with each other (7, 8). It is necessary to have an approach that includes both patient and caregiver needs in order to support AYA patients (9).

Since there is no reproducible AYA program, each pediatric or adult center should create and adapt a dedicated program according to structural and economic resources (5, 10).

Even in Italy, the effort of the Associazione Italiana Ematologia Oncologia Pediatrica (AIEOP) was oriented to the break down AYA’s poor access to pediatric referral centers and their poor inclusion in clinical trials that caused limited improved survival compared to different age patients (11, 12).

Some centers in Italy were pioneers of the AYA-dedicated program, such as the Youth Area in Aviano and the Youth Project in Milan (13), set in two large adult National Cancer Institutes.

In this paper, we discuss how we created an AYA program in a children’s hospital.

## How to Realize an AYA Project in a Children’s Hospital

### The Clinical Aspects

Until 2010, Bambino Gesù Children’s Hospital was the main pediatric hospital in Italy treating patients younger than 15 years of age, and it was thus considered “child-friendly”. Since then, the age limit has been eliminated. From 2015 to 2017, 731 patients were admitted between 0 and 21 years, 239 aged 15–21 years (80% in the range 15–18 years old), representing 32.7% of patients. Currently, each year we manage almost 70 patients up to 25-year-olds. Patients ranging from 18 to 25 years old have been arbitrarily identified in this paper as younger adults.

Lymphomas, sarcomas, and germinal tumors were the main diagnoses in the AYA group. We assisted an increasing number of adolescents and younger adults admitted to our department in a relatively short period, with a consequential impact on the hospital. Looking at the experience of other centers and recognizing the position of the AIEOP, we wanted to carry out a project dedicated to AYA at our hospital. For this reason, in 2017, the oncologists together with the health management staff defined the strengths, weaknesses, opportunities, and threats (SWOT) to create such a project (**Table 1**).

**Table 1:** SWOT evaluation.

Points of weakness	Strengths
Lack of dedicated and structured shelter rooms for adolescents Lack of common areas used for recreation, support, and socialization Multidisciplinary team No dedicated age-adapted PEWS age-adapted TV channels not suitable for adolescents Lack of events or proposals of interest to the adolescent • Lack of volunteering able to relation the adolescent	High care capacity Dedicated multidisciplinary team easy to achieve through internal resources High accessibility to clinical trials Available musculoskeletal oncology program in partnership with the National Cancer Institute in Rome Adolescent units for chronically ill patients (cardiac diseases, kidney diseases, and cystic fibrosis) active in the hospital.
**Threats**	**Opportunity**
Difficulty in rotating adolescent patients in dedicated rooms due to high patient turnover. Difficulty in setting up a leisure room or an easy room because of space capacity. **Lack of external perception “Hospital treats adolescents?”** **Decreased financial support**	Development of an adolescent care that can be reproduced and exported to other pediatric centers AYA-program sustained by the Hospital **Press campaign in which patients promote social and non-scientific communication offered to adolescents and families/institutions** **Increasing fundraising for research and activities from external partners**
**Points addressed by 4YouLab**	Being a testimonial for the press campaign to raise awareness on cancer patients Participating and organizing fundraising for research activities supported by external partners• Promoting partnerships with other pediatric centers.
Organizing schooling, support, and socialization projects. Planning events or proposals of interest for the adolescents Establishing a group of volunteers able to create a stable relationship with the adolescents Finding solutions to refurbish and readapt common areas	

The clinical aspects mainly concerned the management of adolescent/young patients, which differed from children, who needed direct attention in terms of clinical support, communication skills, and caregiver relationship.

The impact of this population on the already existing oncology team was relatively moderate. Through internal seminars and training programs, the oncology, medical and nursing staff acquired the “know how” to treat AYA, drawing from other adolescent dedicated teams operating in the hospital for patients with chronic diseases (cardiac disease, kidney disease, and cystic fibrosis).

This rebuilt team includes professionals such as a pediatric onco-hematologist, nurses, physiotherapists, psychologists, cardiologists, endocrinologists, teachers, social assistants, youth workers, and spiritual assistants.

The partnership with the National Cancer Institute in Rome was already a part of the program for the treatment of musculoskeletal diseases, and then extended to other adult cancer type support. Finally, being a research hospital, the existence of a clinical trial unit granted the accessibility to international trials also for AYA.

### The Psycho-Social Aspects

From the SWOT, several weaknesses were attributable to the lack of structural aspects (hospital setting, environments, rooms, location space, TV channels) and the need of support (psycho-social support, activities and volunteering) for the patients. The first action taken was to focus on an age-oriented project with particular attention to the perceived needs of this population of patients.

We empirically used the anonymously self-administered questionnaire (Needs Evaluation Questionnaire, NEQ Tamburini) already in use at our hospital, to obtain information and communication needs, assistance and care needs, material and economic needs, psycho-emotional needs, and relational needs.

Moreover, we set up in the ward an open diary to register the feedback of the hospitalization setting, the environments, and an evaluation of the service offered, ideas, and suggestions.

In one year (2018), 50 adolescents left their answers and suggestions.

The results showed: 54% desired to spend time with peers during treatment, 56% requested someone who could organize activities for adolescents in the ward, 44% requested someone who could facilitate the relationship between peers in the ward, and finally, 64% expressed the need to have spaces dedicated to age-based activities.

Following these results, we took two steps: employed a youth worker as part of the team (requested figure) and formed a permanent committee (relationship and planning needs), named 4YouLab, with the scope to provide AYA with problem-solving solutions.

The committee members are volunteer adolescent patients, a referred oncologist, two psychologists, and a youth worker, operating to set a “wish list”.

They collaborated to improve the ward setting, the quality of the entertainment offered, the role and purpose of volunteering, and the creation of age-dedicated paths in the department, as well as initiatives and activities aimed at maintaining sociability and normalcy during treatment. This committee acted weekly, driven by the youth worker, while a monthly meeting with the oncologist and psychologists was scheduled as part of the department’s initiatives. Their suggestions were used to improve the quality of the hospitalization offered, with particular attention to the privacy aspects and accommodation in rooms per age.

**Table 1** also shows the SWOT points in which 4YouLab has been involved and the objectives achieved by the committee.

Several projects and workshops were created. The projects were shared on a dedicated website (https://www.facebook.com/foryouobg). Online activities were pursued during the COVID pandemic as a virtual room for adolescents and a remote cine-forum (14, 15).

From this group, some members founded the voluntary association (https://www.4youaps.org/) dedicated to AYA with cancer; the volunteers operate as “expert patients” and are permitted to obtain a volunteer for a specific age groups.

## Discussion

Regardless of how it was made, all proposed models present the limit related to the ability to measure the effectiveness and the benefits provided (16), which is also true for us. It took almost two years to carry out a project in our department dedicated to AYA patients, which involved a restructuring of both the hospital structure, the training of the assistance staff and most importantly, an age-appropriate psychosocial support. For a pediatric hospital with a large flow of patients per year, where everything is oriented towards very young patients (from beds to volunteering), this program was possible due to the SWOT analysis, which measured not only strengths, but also weaknesses, aiding in finalizing the ideas. In our opinion, we would like to highlight for those wishing to start similar projects that an AYA program seems to be feasible in a children’s hospital by starting with the internal and existing resources; in the renewal project, a dedicated “space” and team involving the AYA patient are crucial. There are several projects worldwide focused on the assistance of these subsets of patients, each one respecting the essential rules of clinical and psychosocial nature, but each differs on how to give normality during treatment and to guarantee sociality. The AYA committee was found to be an essential part of the staff of the department, allowing patients to feel a part of an institution, be able to propose and follow projects, be useful, and make improvements not only aimed at their individual care.

However, it is clear that not being able to define the areas of effectiveness is often a limitation for projects similar to ours. In particular, while the clinical aspects such as the care rates and the accessibility to clinical trials can be easily measured, we can only evaluate 4YouLab through direct and indirect anecdotal measurements of its effectiveness.

Directly, we can use the number of participants in the proposed activities, the continuity of the projects, their finalization; indirectly, we can consider the support received outside the hospital and the feedback obtained by caregivers or parents.

In our experience, we assisted, as recognized by the participation to the activities over the years, overcoming the isolation due to treatment and improving the quality of life during treatment. It was attributable to the opportunity to be an active part of a project. These activities, created with our psychologists, were all made with the purpose of exorcising and sharing the fears, taboos, or any kind of wound that the oncological disease has given. We can also affirm that experiences like this can help AYAs to find the necessary resources to cope with the diagnosis path.

Over the years, we have witnessed an improvement of 40% of the requests for meetings with our psychologists directly from the patients themselves, something that had happened very rarely in the past, probably because of the loss of the sense of shame that the disease can cause, loss that matured in the comparison between peers.

We would also like to emphasize the figure of the youth worker, who played a central role in the patient care pathway, representing a cross reference figure between families and caregivers and improving the success of the activities and relationships between peers in the ward. This professional, following patients from day one of their treatment, stimulated the participation to the committee and to the activities. Being alongside adolescent patients on a daily basis facilitates the process of adaptation to hospitalization and treatment from the earliest stages. These favored adaptive coping strategies include peer support and emotional disclosure. Furthermore, the youth worker entered into the treating team with doctors, nurses, and psychologists, reporting critical situations and sharing his experiences with the rest of the team.

However, indirectly, the effectiveness of similar projects can be measured through the support that the project has received in both social and economic terms, and the feedback obtained from patients, caregivers, and various members of the multidisciplinary team.

4YouLab has been invited to several meetings organized by schools and institutes in Rome to show how cancer patients are perceived. This group has also been a testimonial for fundraising events and radio and TV talks; at the local and national levels to raise public awareness and reduce the isolation that cancer can cause.

The benefit of our AYA project, which can be easily reproduced in other contexts, was precisely this: to enable the patients to be both the users and the providers of age-based services and activities. As learned from other experiences, 4YouLab places the adolescents and young adults at the center of the care project, not only in a metaphysical sense, but also in a practical way.

## Author Contributions

GM idealized and coordinated the group. GM, AS, and MI wrote the manuscript. GM and AS performed the literature review. All authors critically reviewed the manuscript and managed the patient. All authors contributed to the article and approved the submitted version.

## Funding

This project was funded by the Progetto Adolescenti 201803X004519 at Bambino Gesù Children’s Hospital, IRCCS.

## Conflict of Interest

The authors declare that the reserch was conducted in absence of any commercial or financial relationship that could be construed as a potential conflict of interest.

## Abbreviations

AYA, adolescent and young adults; SWOT; strengths, weaknesses, opportunities, and threats.
